# Supplementary material for: Current global estimates, risk factors, and knowledge gaps for Hepatitis E virus (HEV): A scoping review
Source: PLoS Negl Trop Dis. 2026 Mar 11;20(3):e0013980. doi: 10.1371/journal.pntd.0013980 (PMC12978505; doi:10.1371/journal.pntd.0013980)
Supplement: S1 File — Systematic database search strategy for 3 databases. (DOCX) [file pntd.0013980.s002.docx]

**S2 File: Full Electronic Search Strategies**

**S5 (A)**

1. Information Sources & Search Summary

- Review Title: Global estimates, risk factors, population at risk and current knowledge gaps in Hepatitis E Virus (HEV): A Scoping Review
- Search Date: January 2024
- Timeframe: Database inception to December 31, 2023
- Language Filter: English
- Database: PubMed

1. Scopus Search Strategy Framework

| Concept | Estimates of Hepatitis E | Risk Factors of HEV Infection | Risk populations for HE and current gaps |
| --- | --- | --- | --- |
| Keywords | estimates, update, epidemiology, seroprevalence, status, synopsis | risk factors | vulnerable populations, current gaps |
| MeSH | "Hepatitis E"[Mesh] OR "Hepatitis E virus"[Mesh]) OR "ORF3 protein, Hepatitis E virus" [Supplementary Concept] OR "ORF2 protein, Hepatitis E virus" [Supplementary Concept] OR "Hepevirus"[Mesh] | "risk factors"[MeSH Terms] OR risk factors[Text Word] | "Vulnerable Populations"[Mesh] |
| Proximity searching | “hepatitis e estimates” [title/abstract:~0] OR “hepatitis e update” [title/abstract:~0] OR “hepatitis e seroprevalence” [title/abstract:~0] OR : “hepatitis e status” [title/abstract:~0] OR “hepatitis e synopsis” [title/abstract:~0] |  | “hepatitis E gaps"[Title/Abstract:~3]) OR "Vulnerable Populations"[Mesh] |
| Search String | “hepatitis e estimates” [title/abstract:~3] OR “hepatitis e update” [title/abstract:~3] OR “hepatitis e seroprevalence” [title/abstract:~3] OR “hepatitis e status” [title/abstract:~3] OR “hepatitis e synopsis” [title/abstract:~3] OR "Hepatitis E"[Mesh] OR "Hepatitis E virus"[Mesh] OR "ORF3 protein, Hepatitis E virus" [Supplementary Concept] OR "ORF2 protein, Hepatitis E virus" [Supplementary Concept] OR "Hepevirus"[Mesh] | Hepatitis e risk factors"[Title/Abstract:~3] OR "risk factors"[MeSH Terms] | ("hepatitis e estimates"[Title/Abstract:~3] OR "hepatitis e update"[Title/Abstract:~3] OR "hepatitis e seroprevalence"[Title/Abstract:~3] OR "hepatitis e status"[Title/Abstract:~3] OR "hepatitis e synopsis"[Title/Abstract:~3] OR "Hepatitis E"[MeSH Terms] OR "Hepatitis E virus"[MeSH Terms] OR "orf3 protein hepatitis e virus"[Supplementary Concept] OR "orf2 protein hepatitis e virus"[Supplementary Concept] OR "Hepevirus"[MeSH Terms] OR "Hepatitis e risk factors"[Title/Abstract:~3] OR "hepatitis E vulnerable populations"[title/abstract:~3] OR "hepatitis E gaps"[Title/Abstract:~3]) AND (english[Filter]) |

1. Full Executable String

("hepatitis e estimates"[title/abstract:~3] OR "hepatitis e update"[title/abstract:~3] OR "hepatitis e seroprevalence"[title/abstract:~3] OR "Hepatitis E"[Mesh] OR "Hepatitis E virus"[Mesh] OR "ORF3 protein, Hepatitis E virus"[Supplementary Concept] OR "ORF2 protein, Hepatitis E virus"[Supplementary Concept] OR "Hepevirus"[Mesh] OR "Hepatitis e risk factors"[title/abstract:~3] OR "risk factors"[MeSH Terms]) AND (english[Filter])

**S2 File: Full Electronic Search Strategies**

**S5 (B)**

1. Information Sources & Search Summary

- Review Title: Global estimates, risk factors, population at risk and current knowledge gaps in Hepatitis E Virus (HEV): A Scoping Review
- Search Date: January 2024
- Timeframe: Database inception to December 31, 2023
- Language Filter: English
- Databases: Scopus

1. Scopus Search Strategy Framework

| Component | Concept 1: Estimates of Hepatitis E | Concept 2: Risk Factors of HEV Infection | Concept 3: Risk Populations & Gaps |
| --- | --- | --- | --- |
| Objective Alignment | Global disease burden estimates (RQ1) | Factors for HEV infection (RQ2) | Vulnerable groups & knowledge gaps (RQ3 & RQ4) |
| Keywords | estimates, update, epidemiol*, seroprevalen*, status, synopsis | "risk factor*" | "vulnerable population*", "knowledge gap*" |
| Proximity Searching (Syntax) | TITLE-ABS-KEY("Hepatitis E" W/3 estimate*) OR TITLE-ABS-KEY("Hepatitis E" W/3 update*) OR TITLE-ABS-KEY("Hepatitis E" W/3 seroprevalen*) | TITLE-ABS-KEY("Hepatitis E" W/3 "risk factor*") | TITLE-ABS-KEY("Hepatitis E" W/3 "knowledge gap*") OR TITLE-ABS-KEY("Hepatitis E" W/3 "vulnerable population*") |

1. Full Executable String

(TITLE-ABS-KEY("Hepatitis E" OR "HEV" OR "Hepevirus") W/5 (estimate* OR update* OR seroprevalen* OR epidemiol* OR status OR synopsis OR "risk factor*" OR "vulnerable population*" OR "knowledge gap*")) AND (LIMIT-TO (LANGUAGE, "English"))

**S2 File: Full Electronic Search Strategies**

**S5 (C)**

1. Information Sources & Search Summary

- Review Title: Global estimates, risk factors, population at risk and current knowledge gaps in Hepatitis E Virus (HEV): A Scoping Review
- Search Date: January 2024
- Timeframe: Database inception to December 31, 2023
- Language Filter: English
- Databases: Web of Science

1. Scopus Search Strategy Framework

| Component | Concept 1: Estimates of Hepatitis E | Concept 2: Risk Factors of HEV Infection | Concept 3: Risk Populations & Gaps |
| --- | --- | --- | --- |
| Objective Alignment | Global disease burden estimates | Factors for HEV infection | Vulnerable groups & knowledge gaps |
| Keywords | estimates, update, epidemiol*, seroprevalen*, status, synopsis | "risk factor*" | "vulnerable population*", "knowledge gap*" |
| Proximity Searching (Syntax) | TITLE-ABS-KEY("Hepatitis E" W/3 estimate*) OR TITLE-ABS-KEY("Hepatitis E" W/3 update*) OR TITLE-ABS-KEY("Hepatitis E" W/3 seroprevalen*) | TITLE-ABS-KEY("Hepatitis E" W/3 "risk factor*") | TITLE-ABS-KEY("Hepatitis E" W/3 "knowledge gap*") OR TITLE-ABS-KEY("Hepatitis E" W/3 "vulnerable population*") |

1. Full Executable String

(TS=("Hepatitis E" OR "HEV" OR "Hepevirus") NEAR/5 (estimate* OR update* OR seroprevalen* OR epidemiol* OR status OR synopsis OR "risk factor*" OR "vulnerable population*" OR "knowledge gap*")) AND LA=(English)
