# Supplementary material for: Current global estimates, risk factors, and knowledge gaps for Hepatitis E virus (HEV): A scoping review
Source: PLoS Negl Trop Dis. 2026 Mar 11;20(3):e0013980. doi: 10.1371/journal.pntd.0013980 (PMC12978505; doi:10.1371/journal.pntd.0013980)
Supplement: Attachment — Submitted filename: Response to Reviewers.docx [file pntd.0013980.s006.docx]

**Reviewers' Comments:**

Reviewer's Responses to Questions

**Key Review Criteria Required for Acceptance?**
As you describe the new analyses required for acceptance, please consider the following:
**Methods**
-Are the objectives of the study clearly articulated with a clear testable hypothesis stated?
-Is the study design appropriate to address the stated objectives?
-Is the population clearly described and appropriate for the hypothesis being tested?
-Is the sample size sufficient to ensure adequate power to address the hypothesis being tested?
-Were correct statistical analysis used to support conclusions?
-Are there concerns about ethical or regulatory requirements being met?

Reviewer #1: The abstract describes the answers to the questions, but the inclusion criteria are not identified.

**Response:**

We really appreciate this feedback. We have updated our abstract with the selection criteria based on PCC framework. We have also incorporated few additional sentences based on other comments. (See abstract line 27-49).

The introduction focuses on items with no direct association with the research problem, and there is no clear rationale for the review; that is, it does not describe why a review with these characteristics is relevant.

**Response:**

We thank the reviewer for this constructive feedback. We agree that the introduction should more explicitly state the rationale for choosing a scoping review methodology. We have revised the final paragraph of the Introduction to clearly articulate the 'knowledge gap'—specifically the fragmented and heterogeneous nature of current HEV burden data. We have emphasized that a scoping review is the most appropriate method to map this broad and diverse evidence base to identify specific gaps that hinder policy development and vaccine implementation. (See Introduction, Page 5, Lines 104-116)."

Reviewer 1

The inclusion criteria are unclear. In addition to a description of the PCC framework, more specific criteria for included studies should be included. Exclusion criteria include publications that were not peer-reviewed, but in the search strategy, the authors state that they also reviewed gray literature.

**Response:**

We appreciate this observation. We have clarified the inclusion criteria by explicitly listing the study designs (including primary research and secondary syntheses) and population subgroups. Regarding grey literature, we have corrected the contradiction: we included authoritative grey literature (e.g., WHO reports, IHME data) while excluding non-authoritative sources (e.g., news blogs, personal opinion pieces). (See Method section, page 9, line 181-199)

Reviewer #2: Major Revision.
This scoping review addresses an important and underexplored topic: the global burden and epidemiology of Hepatitis E virus (HEV), including genotype distribution, seroprevalence, and outbreak occurrence across WHO regions. The topic is of clear public health relevance and aligns with the current WHO priorities for viral hepatitis elimination.
However, despite the study’s potential and the authors’ effort to follow recognized guidelines, the manuscript requires substantial methodological clarification and better alignment with the Joanna Briggs Institute (JBI) framework and the PRISMA-ScR recommendations. Several aspects of transparency, reproducibility, and reporting standards need improvement before the manuscript can be considered for publication.
1. Alignment with JBI and PRISMA-ScR Frameworks
While the authors mention that the review followed the JBI and PRISMA-ScR recommendations, this adherence is not adequately demonstrated.

**Response:**

The Methods section has been entirely restructured into the JBI five-step framework: (1) Identify Research Question, (2) Identify Relevant Literature, (3) Select Literature, (4) Chart Data, and (5) Collate/Summarize Results. We have also explicitly stated that Step 6 (Consultation) was treated as optional per JBI guidelines.

The entire Methods section (Pages 6-12, line 127-266) now follows this structure. The PRISMA-ScR checklist is now included in **Supplementary Material as S1 Checklist**. Respective legend has been included in page 79, line 2049-2051.

- Please explicitly describe each methodological step according to the JBI framework: defining the objectives and review questions, specifying inclusion/exclusion criteria using the PCC structure, explaining data charting, and describing how evidence was collated and summarized.

**Response:**

We have restructured the Methods section to align strictly with the JBI five-step framework for scoping reviews. The following updates were made:

- PCC Framework: We reformatted the inclusion criteria using the Population, Concept, and Context (PCC) structure. Explicit inclusion/exclusion criteria are now detailed on Page 8–9, Lines 181–199.
- Data Charting: The data charting process has been clarified, including the details of the pilot-testing (pilot-testing the charting matrix) to ensure inter-rater reliability. See Page 9-10, Lines 201–225.
- Collation and Summary (Step 5): We have expanded this section to describe our three-stage analytic approach for collation, summarization, and reporting. This includes our descriptive synthesis and the Evidence Maturity Index. See Page 10–12, Lines 227–274.

- Include the PRISMA-ScR checklist as supplementary material, indicating the page or section where each item is addressed.

**Response:**

We have provided an updated PRISMA-ScR Checklist (S1 Checklist) as supplementary material. This document identifies the specific page and section numbers within the clean version of the revised manuscript where each of the 22 reporting items is addressed.

The two-step search strategy is comprehensive and demonstrates a commendable effort to capture both indexed and gray literature. However, a few aspects compromise reproducibility and methodological transparency:
- The complete search syntaxes should be presented for all databases, not only for PubMed. The current mention of “syntax available in supplementary data” is acceptable but should include a clear summary in the main text.

**Response:**

For better clarity and transparency, we have presented a clear summary of all three-search strings in the main text using a table. In addition, complete search strategy for all 3 databases have been included in supplementary file as S5 Table in page 79.

- The use of Google Scholar for country-specific searches is innovative but must be better justified and methodologically standardized. Please explain how the first 100 results or the first 10 pages, for example, were screened to avoid selection bias.

**Response:**

We have standardized and justified the Google Scholar search by screening the first 50 results per country to ensure reproducibility and mitigate indexing bias. (See page 7 line 163-166)

- Clarify whether the searches were restricted to English only and discuss how this may have influenced geographic representation (language bias).

**Response:**

We have updated the Methods (Page 8, Line 173-174) to explicitly state the English-language restriction. To address the reviewer’s concern regarding language bias, we have added a dedicated paragraph in the Discussion/Limitations section (Page 45, line 825-831). This paragraph explores how the exclusion of non-English sources (e.g., French, Spanish, Chinese, or Portuguese) may have contributed to the "Data Desert" findings in specific regions, even though we utilized international surveillance reports to mitigate this gap.

The PCC structure is appropriate, but its application could be better defined:
- Consider refining the Concept domain: currently, it mixes “factors of risk” and “burden estimates” without clear prioritization. Define whether the main outcome is HEV burden, risk factors, or knowledge gaps.

**Response:**

We appreciate the reviewer’s prompt to clarify the conceptual hierarchy. While scoping reviews traditionally aim to map a broad field rather than isolate a single clinical outcome, we recognize the importance of defining the review’s ultimate objective.

We have refined the Concept domain to clarify that the primary synthesis outcome is the identification of global knowledge gaps. In this framework, data on HEV burden, risk factors, and high-risk populations are treated as the thematic domains required to reach that synthesis. This hierarchy ensures that the review does not merely list disparate data points but integrates them to reveal the Knowledge Gaps. (See page 9, line 181-193).

- Clarify the inclusion of different study designs (cross-sectional, case-control, cohort, surveillance reports) and how these were treated in the synthesis.

**Response:**

We have provided a detailed clarification of our approach to study designs in the Methods (Page 10, Lines 206-221). Given the heterogeneity of the 395 included articles, we developed a hierarchical string-matching taxonomy to categorize evidence into five functional groups (Evidence Synthesis, Surveillance/Public Health, Observational–Analytical, Experimental/Laboratory-based, and Observational–Descriptive). We have presented the descriptive analysis of this in table 2 (Page 19).

- Discuss whether modeling studies or systematic reviews reporting HEV burden estimates were included or excluded, as this affects comprehensiveness.

**Response:**

We have clarified the inclusion of these study types in the Methods (Page 9, Line 193-196). To ensure maximum comprehensiveness, we included both modeling studies and systematic reviews. By including these sources, we were able to compare "modeled" burden against "observed" empirical data, which was a critical component in identifying geographic "Data Deserts" where empirical data are lacking but modeled burden is high.

The use of Rayyan for citation management and double-blind screening is a strength and aligns with good review practices. However:
- Specify how inter-reviewer agreement was measured (e.g., Cohen’s kappa) and what steps were taken to resolve discrepancies.

**Response:**

We acknowledge the reviewer’s request for clarification on inter-rater reliability. While formal statistical measures such as Cohen’s Kappa are not a mandatory requirement for scoping reviews under the JBI framework, we ensured a high level of screening consistency through a rigorous consensus-based approach.

All titles, abstracts, and full texts were screened in a double-blind manner. Any discrepancies were resolved through direct discussion among three 3 reviewers until 100% agreement was reached. For any disagreement which was not resolved by discussion was adjudicated by a senior reviewer. This process aligns with the JBI Manual for Evidence Synthesis. Please see page 8, line 176-178.

- The manuscript mentions the use of a priori structured matrices for data extraction. Please describe their structure (variables collected, pilot testing, consistency checks).

**Response:**

We have expanded the Data Extraction section (Page 9, Line 201-206) to provide a detailed description of the extraction matrices. The *a priori* matrix was developed based on the JBI data extraction template and customized to capture HEV-specific variables. To ensure the tool's reliability, we conducted pilot testing on a subset of 10 articles, which led to the refinement of variable definitions.

- Clarify whether data extraction was duplicated independently or performed by one reviewer and checked by another.

**Response:**

Data extraction was based on collaborative independent extraction among the reviewers. As detailed in the Methods (Page 9-10, line 201-206), three reviewers (MKA, HM, MB) independently extracted data from the 395 included studies into a standardized Excel matrix. At regular intervals the extraction was further reviewed and discussed by the senior reviewer.

The manuscript describes the extraction of key information (authors, year, genotype, seropositivity, outbreak data), but the analytical strategy remains vague:
- Scoping reviews do not usually perform meta-analysis, but they do require a clear narrative synthesis framework — e.g., grouping by region, study type, or outcome category.

**Response:**

We really appreciate your feedback. Based on this comment we have significantly expanded the analytical approach (See page 11-12, line 227-274). We move beyond simple data listing to a **structured three-stage synthesis**:

- Descriptive analysis of studies by frequency and counts on several key characteristics (Page
- Global, regional and country-specific analysis
- Two stage analytical approach for risk factor and gap analysis. Following previous studies approaches we have applied Evidence Maturity Index and Regional Discordance Mapping to highlight the gap and identify the Priority Blind Spots.

- Describe how findings were summarized (frequency counts, geographical mapping, identification of gaps).

**Response:**

As explained in the last comment, we have used frequency counts to describe the characteristics of 395 studies in terms of year of publication, study design, WHO region, population and genotype etc. Please see table 2 in page 19. For gap analysis, we used two stage approach using Evidence Maturity Index and Discordance mapping. We have explained in detail in the method section step 5 in page 10-12. We have also presented results using specific figures (Figure 3: Global HEV Evidence Maturity by WHO Region in page 39, line 698-702; Figure 4: Global Discordance: Disease Burden vs. Evidence Maturity in page 40, line 712-717; and Figure 5: Regional Virological Blind Spots & Transmission Profiles in page 41, line 728-731).

- Consider adding quantitative mapping (tables or figures) showing the distribution of included studies per WHO region, genotypes, and main outcomes.

**Response:**

We really appreciate this feedback. We have added additional quantitative mapping using tables and figures. We have used Table 2 in page 19 to show the distribution of included studies per WHO region, genotype, population and reported risk factors. As mentioned in the previous comment, we have also presented results using specific figures (Figure 3: Global HEV Evidence Maturity by WHO Region in page 39, line 698-702; Figure 4: Global Discordance: Disease Burden vs. Evidence Maturity in page 40, line 712-717; and Figure 5: Regional Virological Blind Spots & Transmission Profiles in page 41, line 728-731).

Finally, the objective is clear, but there is an absence of an explicitly formulated hypothesis.

**Response:**

We really appreciate this feedback. Although formulation of explicit hypothesis is not a mandatory requirement of the JBI framework for scoping review, we have explicitly mentioned our working hypothesis in method section step 1 (Page 6, line 137-138).

**Results**


-Does the analysis presented match the analysis plan?

-Are the results clearly and completely presented?

-Are the figures (Tables, Images) of sufficient quality for clarity?

Reviewer #1: The results are consistent with the methodology and are clearly presented; however, the previous comments should be taken into account. The tables are adequate and provide sufficient information.

**Response:**

We are very thankful to you. Your previous comment regarding restructuring the method section as per the JBI step wise framework has been addressed. The entire Methods section (Pages 6-12, line 127-266) now follows this structure.

Reviewer #2: Major Revision.

The authors conducted an extensive review of the international literature on the global epidemiology of hepatitis E. However, the data included in the manuscript are somewhat outdated, and it would be advisable to insert comments in the discussion section to reflect more recent evidence.

For example, the search period for the studies included in this review extended up to December 2024. Nevertheless, recently published data from Latin America, particularly from Brazil, indicate that the prevalence of hepatitis E varies widely. The overall prevalence of the IgG marker in Brazil ranged from 0.5% in the North region to 59.4% in the South region (Mariz et al., 2025). However, in the present review, the authors present a prevalence ranging from 1.7 to 38%, somewhat divergent from what is available in the literature. Similar updated findings have been reported in several other parts of the world.

It is therefore strongly recommended that the authors update the references and include recently published and relevant studies on the hepatitis E virus, such as:

Magri, M. C.; Manchiero, C.; Dantas, B. P.; Bernardo, W. M.; Abdala, E.; Tengan, F. M. Prevalence of hepatitis E in Latin America and the Caribbean: A systematic review and meta-analysis. Public Health 2025, 244, 105745.

Songtanin, B.; Molehin, A. J.; Brittan, K.; Manatsathit, W.; Nugent, K. Hepatitis E Virus Infections: Epidemiology, Genetic Diversity, and Clinical Considerations. Viruses 2023, 15, 1389.

Mariz, C. A., de Araújo, L. R. M. G., & Lopes, E. P. (2025). Hepatitis E Virus Infection in Brazil: A Scoping Review of Epidemiological Features. Pathogens (Basel, Switzerland), 14(9), 895. <https://doi.org/10.3390/pathogens14090895>

**Response:**

We thank the reviewer for highlighting these recent and highly relevant publications. We acknowledge that the HEV epidemiological landscape is rapidly evolving, particularly in regions like Latin America. We have added into the discussion about these limitations. However, a cutoff is necessary in order to analyze the results and synthesize the discussion.

In addition, the tables should be revised to ensure that they are presented in an appropriate format and that references are clearly indicated when the author is cited.C

**Response:**

We have expanded the "Limitations" section to clarify that while our systematic search concluded in December 2024, the findings from these 2025 publications validate our study's identification of high-variability zones and the need for continuous evidence monitoring. We have acknowledged the limitation in discussion section page 45 line 840-844.

**Conclusions**


-Are the conclusions suplimitationssthe data presented?

-Are the limitations of analysis clearly described?

-Do the authors discuss how these data can be helpful to advance our understanding of the topic under study?

-Is public health relevance addressed?

Reviewer #1: The conclusions are consistent with the research questions and the objective of the study. The discussion clearly describes the limitations, scope, and significance of the study, as well as the relevance of the results in the context of public health.

Reviewer #2: Limitations: Adequate transparency but requires a more analytical and updated discussion of the consequences of methodological restrictions.

**Response:**

We have revised the Limitations section to move beyond a description of our restrictions to an analytical assessment of their consequences. We explicitly discuss how language bias may mischaracterize evidence gap and global estimates and how our 2024 temporal cut-off serves as a conservative baseline. Please see page 45, line 827-832 and line 840-846.

Excerpt analyzed:

“...this review is limited by the selection bias related to the use of bibliographic citations from 3 databases... we did not incorporate the information from any resources not published in English... Additionally, we did not conduct critical appraisal of the included studies.”

Evaluation:
The limitations are clearly identified and reflect transparency in reporting, which is commendable. The authors appropriately acknowledge potential selection bias due to the restriction to three major databases and to English-only publications. However, the discussion of limitations is too brief and lacks depth in terms of methodological implications.

Points for improvement:

The authors should quantify or qualify the impact of these limitations. For instance, how significant might the exclusion of non-English studies be, particularly in regions such as Latin America, Africa, and Asia, where HEV epidemiology is less represented in English-language literature?

**Response:**
We have acknowledged and articulated the impact of these limitations in the discussion section. We have clarified that such limitations may lead to underestimated disease burden estimates particularly in regions such as Latin America, Africa, and Asia, where HEV epidemiology is less represented in English-language literature.

The absence of a critical appraisal (risk of bias assessment) should be further justified. While scoping reviews do not always require formal quality assessment, it is advisable to acknowledge the potential heterogeneity and uneven quality of the included studies and discuss how this may affect the reliability of synthesized evidence.

**Response:**

We have expanded the justification for the absence of a formal critical appraisal in the Limitations section. Following JBI and PRISMA-ScR guidelines, we prioritized the **breadth of evidence mapping** over a quality-weighted synthesis of clinical outcomes. We explicitly acknowledge that this choice results in high heterogeneity due to variations in assay sensitivity and case definitions across the 395 studies (Page 45, line 833-840).

The authors could also mention the temporal limitation of the search (until December 2024) and note that recent studies have since been published, some of which might alter the global prevalence estimates, including new data from Latin America (e.g., Mariz et al., 2025; Magri et al., 2025).

**Response:**

We thank the reviewer for this important point. We have addressed the temporal limitation of our search in the Discussion (Page 45, line 841-847).

Conclusion: Conceptually solid and aligned with the study’s aims, but requires refinement in structure and specificity to enhance analytical depth and scientific rigor.

Excerpt analyzed:

“The study systematically reviewed global evidence... found a severe lack of data on HEV incidence and mortality... highlighted potential risk factors... stakeholders should prioritize routine testing and vaccination strategies.”

Evaluation:
The conclusion is coherent and consistent with the objectives of the study. It successfully summarizes the key findings and emphasizes the public health relevance of HEV.

**Response:**

We thank the reviewer for this constructive feedback. We have refined the Conclusion to more explicitly bridge our core data with our final synthesis. By quantifying regional gaps—notably the 51.4% "Absolute Desert" rate in the Western Pacific—and applying our specific frameworks of "Discordance Mapping" and "Molecular Blind Spots," we have shifted the narrative from a general summary to a precise analytical roadmap (Page 46, line 851-854).

Positive aspects:

The conclusion connects findings to policy and public health implications, particularly the need for equitable vaccination strategies.
It underscores the global neglect of HEV and the variability of existing data, an important contribution to the literature.

**Response:**

We are very thankful to the reviewer for such positive comments.

Points needing improvement:

The conclusion could differentiate better between what was found and what is recommended. Currently, both are intertwined, reducing the analytical clarity. For instance, the statement “our review highlighted potential risk factors which need to be well understood for future policies” should specify which risk factors (e.g., zoonotic exposure, sanitation deficits, immunosuppression).

**Response:**

We have restructured the Conclusion to first delineate our empirical findings—specifically citing identified risk factors such as environmental sanitation deficits, zoonotic pork-related exposures, and host-specific vulnerabilities (immunosuppression and pregnancy). We have then separated these findings from our strategic recommendations to ensure a more rigorous transition from evidence to policy. Please see page 46-47, line 849-876.

The text could also benefit from greater precision and cautious language regarding causality, avoiding phrases that imply direct evidence (e.g., “need to be well understood”) when the review primarily identifies knowledge gaps.

**Response:**

We have revised the manuscript to ensure more cautious language regarding causality, specifically in the Discussion and Conclusion. We have replaced directive phrases with more calibrated academic hedging; for instance, changing "need to be well understood" to "future investigation to clarify their role in transmission." Please see page 46, line 863-865.

The paragraph might end with a forward-looking sentence emphasizing the need for standardized global data collection frameworks, harmonized surveillance, and integration of HEV testing in routine diagnostics.

**Response:**

We have revised the last paragraph with a forward-looking sentence. Please see line 867-873.

Suggested refinement (example excerpt):

“Future research should prioritize the generation of standardized and comparable data across regions, including the systematic integration of HEV testing in routine surveillance. Strengthening global datasets will be critical for guiding vaccination strategies and reducing the burden of this neglected, yet vaccine-preventable, disease.”

**Editorial and Data Presentation Modifications?**


Use this section for editorial suggestions as well as relatively minor modifications of existing data that would enhance clarity. If the only modifications needed are minor and/or editorial, you may wish to recommend “Minor Revision” or “Accept”.

Reviewer #1: In the methodology, the authors should clarify the inclusion criteria and in the introduction they should clearly describe why this review should be conducted.

**Response:**

We have addressed this feedback. (See Method section, page 9, line 186-192)

Reviewer #2: Major Revision.

**Summary and General Comments**


Use this section to provide overall comments, discuss strengths/weaknesses of the study, novelty, significance, general execution and scholarship. You may also include additional comments for the author, including concerns about dual publication, research ethics, or publication ethics. If requesting major revision, please articulate the new experiments that are needed.

Reviewer #1: The review could contribute to the knowledge on the global epidemiology of HEV, but I have some concerns about its validity and reliability.

**Response:**

We appreciate the reviewer’s caution regarding the validity and reliability of the synthesis. We have revised and expanded the method section addressing the issues related to validity and reliability, shared by the reviewers.

Reviewer #2: This manuscript addresses an important and often neglected topic in global health — the epidemiology and disease burden of Hepatitis E virus (HEV). The authors have undertaken a comprehensive scoping review that compiles and synthesizes the global evidence base on HEV prevalence, risk factors, and populations at risk for severe infection. The topic is highly relevant and timely, considering the limited attention HEV receives in public health agendas and the lack of reliable global data on incidence and mortality. The review has the potential to make a valuable contribution to the literature and to inform future research and policy priorities.

**Response:**

We sincerely thank the reviewer for their positive assessment of the study’s relevance and its potential to inform global health policy.

However, before the manuscript can be considered for publication, substantial revisions are needed to improve analytical depth, methodological transparency, and interpretative precision. The current version would benefit from a more critical discussion of its methodological limitations, particularly regarding language restriction (English-only studies), database coverage, and the absence of critical appraisal of included studies. These aspects could introduce systematic bias and should be more explicitly addressed in the Limitations section.

**Response:**

We thank the reviewer for this critical guidance. We have significantly expanded the **Limitations** section to provide a more transparent and critical assessment of methodological boundaries.

In the Conclusion, the manuscript successfully summarizes the main findings and highlights policy implications. Nonetheless, it should more clearly differentiate between observed results and recommended actions, providing greater specificity about the key risk factors identified and the nature of evidence supporting each one. Strengthening the analytical clarity and including a forward-looking perspective emphasizing the need for standardized global HEV surveillance would increase the manuscript’s impact and policy relevance.

**Response:**

We thank the reviewer for this guidance. We have restructured the Conclusion to clearly differentiate empirical Findings from strategic Recommendations (See page 46, line 851-876). To increase specificity, we explicitly named the identified risk domains—sanitation deficits (45.1%), zoonotic pathways (pork-related), and host-specific vulnerabilities—and adopted more cautious language regarding causality (e.g., "offers a pathway to"). This refinement ensures the conclusion is directly anchored in our evidence mapping while providing a high-impact, forward-looking perspective on global surveillance.

In summary, this is a relevant and promising manuscript that addresses an important gap in the global understanding of HEV. I recommend major revision to address the issues noted above, refine the methodological and interpretative sections, and ensure the conclusions are well supported by the evidence synthesized. After these revisions, the study would be a strong candidate for publication.

**Response:**

We sincerely thank the reviewer for the encouraging feedback and the constructive critique. We have undertaken a major revision of the manuscript, specifically refining the Methodology to include an expanded analytical framework and additional quantitative mapping. By applying the Evidence Maturity Index (EMI) and Discordance Mapping, we have significantly enhanced the rigor and interpretative depth of our findings. We believe this refined approach not only addresses the identified gaps but also provides a robust methodological template for evidence mapping in other infectious disease domains.

PLOS authors have the option to publish the peer review history of their article ([what does this mean?](https://track.editorialmanager.com/CL0/https:%2F%2Fjournals.plos.org%2Fplosntds%2Fs%2Feditorial-and-peer-review-process%23loc-peer-review-history/1/010f019a6f007920-b8370e38-7e10-439d-ae7a-2542148a33c6-000000/sXQjIzPPbDApo-eCMGzR2xMl8O_1xirwcfnQKXSAEzM=236)). If published, this will include your full peer review and any attached files.


If you choose “no”, your identity will remain anonymous but your review may still be made public.


**Do you want your identity to be public for this peer review?** For information about this choice, including consent withdrawal, please see our [Privacy Policy](https://track.editorialmanager.com/CL0/https:%2F%2Fwww.plos.org%2Fprivacy-policy/1/010f019a6f007920-b8370e38-7e10-439d-ae7a-2542148a33c6-000000/Dla8tefo4e3seNFoobuAYVNU8gv9R71X7NhUNEu4Osk=236).

Reviewer #1: No

Reviewer #2: No

[NOTE: If reviewer comments were submitted as an attachment file, they will be attached to this email and accessible via the submission site. Please log into your account, locate the manuscript record, and check for the action link "View Attachments". If this link does not appear, there are no attachment files.]
